# Inclusion in the university: Who assumes responsibility? A qualitative study

**DOI:** 10.1371/journal.pone.0280161

**Published:** 2023-01-20

**Authors:** María José Solis-Grant, María José Bretti-López, Camila Espinoza-Parçet, Cristhian Pérez-Villalobos, Iván Rodríguez-Núñez, Cristian Pincheira-Martínez, Cristóbal Sepúlveda-Carrasco

**Affiliations:** 1 Kinesiology Department, School of Medicine, Universidad de Concepción, Concepción, Biobío, Chile; 2 Universidad de Concepción, Concepción, Biobío, Chile; 3 Direction of Teaching, Universidad de Concepción, Concepción, Biobío, Chile; 4 Medical Education Department, School of Medicine, Universidad de Concepción, Concepción, Biobío, Chile; 5 Universidad de Las Américas, Concepción, Biobío, Chile; University of Foggia: Universita degli Studi di Foggia, ITALY

## Abstract

Society challenges higher education institutions and their members to generate inclusive communities to enable the full development of all members. This study aims to analyze who is responsible for generating inclusion according to community members from a traditional Chilean University. We carried out qualitative research based on the Grounded Theory. We collected data through focus group and semi-structured Interviews, involving 14 undergraduate students, two post-graduate students, 17 faculty members, five non-teaching staff members, and nine executives officers. All of thembelonging to the three campuses of the University. We analyzed data using ATLAS.ti 7.5.7, using the constant comparison method and reaching an axial codification level. From the data analysis, 25 subcategories emerged, grouped into six categories. Later we organized them under the codification paradigm. Results highlighted the perception of the interaction and influence of the social, institutional, and personal fields in the inclusion phenomenon. Also, that inclusive practices must be a responsibility shared among different educational community members.

## Introduction

At a time of accelerated social changes, inclusion in education plays a fundamental role in thinking about a more sustainable development model [1]. Inclusion in education is a right recognized by various international and national legal instruments, which has led to the generation and improved of policies that ensure the quality of education and non-discrimination, seeking to promote the access, permanence, and successful completion of the education process for all students, regardless of their particular characteristics [2]. This scenario challenges higher education institutions to develop inclusive communities [3] and provide an equitable educational offer that responds to the needs and interests of the students, thus allowing all students to achieve their maximum level of development and learn about their abilities [4].

For universities to be inclusive, they need to assume commitments from institutional management that have a cross-cutting impact, not only on students but also on the entire educational community and all areas, for example, in administrative aspects, accessibility and the implementation of specific actions in the classroom [5, 6]. Furthermore, as in the school environment, universities need to advance in the three dimensions proposed by the Index for Inclusion: Policy, Culture and Practice [7], which have been incorporated in research on inclusion in tertiary education in recent years [8–10]. National and international legal instruments support the need to strengthen the inclusive approach at all levels of education. Some of the documents that stand out are Article 26 of the Universal Declaration of Human Rights [11], the Sustainable Development Goals proposed by the United Nations in its 2030 Agenda [12] and the International Convention on the Rights of Persons with Disabilities [13]. In Chile, this is supported by extensive legislation in social, labor and educational areas [14–16]. There is consensus on the need for more inclusive education and its legal imperative in international and national frameworks. However, there is a lack of clarity about what strategies would be appropriate to promote it in higher education, and what role corresponds to each university community member. In this context, there is a lack of knowledge about the stakeholder responsibility phenomenon for inclusion in higher education. However, a theoretical background is generated from research at different educational levels, which allows the present research to be guided by the following assumptions. Firstly, inclusion is a process of improvement, a system of beliefs and educational practices favoring teaching and learning processes in a common context [17].

Secondly, there is evidence that a positive attitude on the part of teachers and students is fundamental to fostering inclusive environments in higher education [18]. Thirdly, there is consensus on the idea of the educational institution as the core that enables change by promoting an inclusive culture and practices involving all members of its educational community [7]. In other words, the participation of the university institution is a prerequisite for involving all members of its community in the task of inclusion [19].

In this context, Booth and Ainscow’s Index of Inclusion [7], originally designed for schools, has now been used in research that has adapted its domains for application in higher education [8–10, 20]. The three dimensions of the index and its indicators allow us to understand how the phenomenon of inclusion unfolds and, in the case of this study, to elucidate possible associated responsibilities.

The first dimension is culture, which is the shared values that develop within an educational community. The second dimension points to policies, which correspond to the collaborative organizational plans and spaces that, according to the index, bring together in a common framework the modalities of student support but which, in a fully inclusive university, should also involve means of support for teaching, non-teaching, and management staff. The third dimension refers to practices, understood as actions that demonstrate the educational culture and inclusive policies developed within the educational institution [21].

The social responsibility of universities [22] and the legal regulations that govern them make them guarantors of the establishment of inclusive institutional policies that must be translated into concrete indicators, including facilities, services, training, and access to materials, among others [2–23]. Despite the above, most education systems are characterized as entities that promote the segregation of those students who are academically disadvantaged, either because of their socio-economic vulnerability, their ethnic origin, the presence of disability, their gender, or their religion, among others [24]. Although some higher education institutions show evidence of adaptations in methodology, infrastructure, and services for students with disabilities [25], the same group of students has pointed out the existence of barriers in these same areas [26–29]. These have increased with the advent of online education [1], representing an even more significant barrier for students from poorer sectors [30]. In addition, students with sexual or gender diversity have reported the absence or lack of socialization of protocols on harassment or discrimination and the negative impact this has on their experience of university life [31].

On the other hand, in findings within the university context [25], one of the main barriers perceived by students with disabilities is the negative attitude of teachers, who question their students’ abilities, do not adapt their methodological resources, and even doubt the veracity of their disability status [26]. This coincides with other studies in higher education institutions [32] where, although academic staff showed a positive attitude towards disability and valued inclusive education strategies, in theory, they did not implement them in practice. Along these lines, higher education teachers show high-stress levels associated with making curricular adaptations for students with disabilities, as they are not sufficiently prepared to carry them out [19].

In this context, evidence shows that building inclusive educational institutions goes beyond a legal provision and must be translated into shared responsibility and necessary articulation between the different members of the educational community and the institutional structure.

In order to provide empirical and conceptual background to strengthen the process of implementing inclusive guidelines in higher education institutions, this research seeks to analyze how members of the educational community perceive who should take responsibility for generating inclusion in a higher education institution. For this task, we understood responsibility as the individuals’ and organizations’ capacity and obligation to be accountable for their actions and omissions [33].

This study was carried out in the context of the chilean higher education system, distributed among 58 universities, that offer technical and professional degree programs and postgraduate and graduate programs. Of these, 27 receive direct state support and are known as "Traditional Universities," while another 31 are private universities that also receive state support through indirect means, such as scholarships to students [34]. This research was carried out in one of the traditional universities in the country.

## Materials and methods

The Ethics, Bioethics, and Biosafety Committee of the Universidad de Concepción in Chile approved the present research (CEBB 703–2020). It is a qualitative study that address the phenomena from the point of view of the persons involved [35]. We used the Grounded Theory of Strauss and Corbin [36]. This methodology connects the multiplicity of perspectives of the actors around the studied phenomenon and develops theories and concepts based on data compilation and its systematic analysis [36]. The Grounded Theory also provides a systematic methods that supports data abstraction to develop a theory based on empiric data. These methods include different codification procedures based on the constant comparison method [37].

The study was carried out in a traditional university in Chile’s second most populated city. This university receives direct financing from the chilean State and uses the standard student selection process at the national level.

We carried out focus groups and semi-structured Interview for data collection. The meetings were carried out and recorded using the Zoom© online meetings platform, after the prior signature of individual informed consent. During the informed consent process, we explained to the participants: the purpose of the study, the kind of participation requested, the guarantee for free and voluntary participation, the confidentiality of their data, and their right to withdraw without consequences.

Each participant received a written informed consent form before the focus group or interview meeting, including this information. They had to print and sign the form and then submit it to the researchers. However, the researchers verbally repeated the informed consent key topics at the beginning of the focus groups and interviews.

The meetings were facilitated by two skilled psychologists using scripts generated by the research team (S1–S4 Tables).

We used convenience non-probabilistic sampling through open convocation to the academic and administrative staff and graduate and postgraduate students. After that, we used theoretical sampling. Its selection criterion was to include new cases, according to their potential contribution to development and refining the evolving theory. So, we selected participants who could provide information about less developed topics or whose perspectives could differ from those we interviewed before. We selected new cases until reaching a theoretical saturation [37].

Participants belonged to the three campuses of the University, located in three different cities, and from the five groups of the institution: undergraduate and graduate students, faculty members, non-academic staff, and executive staff. For student groups, we considered regular students in the semester of application of the focus groups as an inclusion criterion. For the executives staff, we selected those members that headed a university unit recognized in the organization chart and were in charge of personnel. Regarding the faculty staff, we called those university members under contract to dictate courses in undergraduate and postgraduate programs of the institution. Regarding the non-teaching officials, we included the professional and non-professional staff members contracted by each institution to carry out functions different from teaching.

In the Focus Groups, we included 14 undergraduate students, two postgraduate students, 17 faculties, and one non-teaching official. Nine executive members participated in semi-structured interviews. Concerning gender distribution of the whole sample, 24 (51,06%) were women, 22 (46.81%) were men, and one (2.13%) was non-binary. Two neurodiverse persons participated [38]. Also, in the sample, we had two students with visual disabilities, and one person self-identified as part of the sexual dissidence considered a political and activist stance against the hegemonic heterosexual norm. Concerning knowledge disciplines [39], participating in the sample, we included members from natural sciences, engineering and technology, medical, and health sciences, social sciences, and humanities. Each participant was invited only once to each meeting.

We first carried out an open codification process for data analysis that allowed us to identify and describe categories inductively (S5 Table). We used the ATLAS.ti 7.5.7 qualitative data analysis program [40]. The second analysis stage was an axial codification. We used it to connect the concepts and categories identified in the open codification. We used the codification paradigm [36] to this end, which focuses on and links causal conditions, the context, intervening conditions, action/interaction strategies, and the consequences.

## Results

From the total results obtained in the study, this article focuses on those referring to the responsibility associated with institutional inclusion. These results were presented to the whole community, leaving open space for comments. It was not necessary to modify the results of this article.

### Open codification

From the reading and analysis of the transcribed documents, 25 subcategories emerged, grouped into six categories related to the perception of the responsibility in the institutional inclusion phenomenon. The categories and subcategories are detailed below (Table 1), illustrated by textual quotations extracted from the transcriptions.

**Table 1:** Categories and subcategories open codification.

Categories	Subcategories	Textual quotation
Organizational characterization of the University	The organization has a very large structure and traditional mentality	*“I believe we function here with a very antiquated thought in several areas (…) I believe they had very good ideas for the society of 1919*, *but not for a 2021 society”* [F.G. 2, female executive]
	It is necessary to revise the institutional bases	*“We need to rethink and restate fundamental issues to take inclusion seriously”* [F.G. 3, female teacher]
	We feel listened to, but we do not take part in decision-making	*“We believe that there is the disposition to listen*, *but not necessarily of taking into account the opinion of the University workers”* [F.G. 7, female teacher]
Institutional diversity and inclusion	Inclusion in this University is limited at present, but work is being done about it	*“As I said*, *this University is not inclusive at present*, *but it does work on it*, *it is not stagnant or closed to that possibility”* [F.G. 1, male functionary]
	The University is very diverse	*“It does have a wide diversity of groups*, *persons*, *orientations*: *it is almost incredible to find all this*” [F.G. 1, female student]
	There is a tendency to homogenize the expression of diversity	*“At 3rd year [of career]*, *they are all the same*, *the people even dress alike”* [F.G. 1, female teacher]
	When people belong to a diversity group there is a tendency towards self-exclusion	*“He did not want any additional help because he felt positively discriminated against*, *because he had a hearing problem (*…*) he also had financial resource problems*. *So we wanted to help him but he didn’t want help*, *and he didn’t want help”* [F.G. 4, female ]
	There have been changes in inclusion and institutional diversity	*“We have trans students*, *students from different ethnic groups*. *They seem to be more visible now”* [F.G.1, female executive ]
Conceptualization of Inclusion	What is understood by inclusion?	*“Any person has to be in a similar condition*, *regardless of his preconditioning factors (…) because many people think that inclusion is like making life easier to other people*, *but it is not so*, *I mean… It is to adapt oneself and the establishment also adapt itself to the disability or any type of problem that the student may have (…) that both should have the capacity to adapt themselves”* [F.G. 1, male ]
	It is necessary to review the concepts of inclusion and diversity	*“It is very ambiguous [the inclusion concept] it does not mean anything (…) For whom*, *in what*, *wherefrom*, *what are its limits*, *its hedges”* [F.G. 3, male executive ] *“I believe that we still have a very reductionist vision of what inclusion is*, *very paternalistic*, *very as giving things as this were charities and not as a right”* [F.G. 1, female executive ]
	Who must be subjects of inclusion?	*“Here I do not have a clear idea*, *I not have a clear idea (…)The groups with access difficulties are infinite*, *that’s the truth… So*, *is this University being realistic*? *Up to what point can it be in charge of everyone*?*”* [F.G. 3, female teacher ]
	There have been social changes in inclusion and diversity issues	“*The fact is that these groups that–traditionally werecalled marginalized today beging to be visualized and manifest themselves”* [F.G. 5, male student ] *“But I certainly believe that this [The inclusion] starts from the people*, *from humanity*, *from the external context from which come our young people (…) and finally the university becomes responsible”* [F.G. 1, male executive ]
Perception of responsibility by areas	Assessment of the inclusion responsibility in the infrastructure	*“Although It is a goal of all [inclusive infrastructure]*, *I mean*, *a University goal (…) University should be structurally fit so that the students may participate in class more inclusively””* [F.G. 3, male student ]
	Assessment of the inclusion responsibility in Admission	*“The University must be much more active in detecting those barriers [of admission]*, *not creating them*, *and favor the groups that are not arriving*” [F.G. 2, male teacher ]
	Assessment of the responsibility for inclusion in the Teaching-Learning Process	*“Everything must be very exactly formalized and quite rigid*, *for example*, *you cannot necessarily change that while a subject is ongoing”*[F.G. 6, female teacher ]
	Assessment of the inclusion responsibility in participation	*“I feel that they come [inclusion-related initiatives] from the students rather than from the university"* [F.G. 2, female student ]
Assessment of the institutional diversity support	At present, there are institutional strategies to include	*“In our faculty*, *we have established specific protocols [about inclusion]*. *However*, *I do not know about an inclusion protocol established as a community or academy*.*”* [F.G. 1, female student]
	There are differences in the perception of support according to the type of diversity	*“Yes*, *it is possible to find kinds of inclusion in certain areas (…)*. *If we “Yes*, *it is possible to find different kinds of inclusion in certain areas (…)*. *If we look at other disabilities [other than visual disability]*, *we find absolutely nothing”* [F.G.1, male student ] *“In fact*, *from the gender commissions itself*, *I was told that the Institution does not take the diversities seriously*, *as if these are problems not worthwhile being taken care of”* [F.G. 2, non-binary student ]
	It is necessary to strengthen institutional support and clarify the role of the teachers	*“And the topics of inclusion as a need for the teachers has been appearing for two or three years already”* [F.G.2, male executive ]
Challenges to generate an inclusive Institution	We have the responsibility to transfer the regulations and protocols to personal practices	*“from the declaration[of inclusion] to the fact*, *there must be a process (…) so that all staffs*, *units and the people individually should be able to implement these rules into concrete facts”* [I1, female executive]
	The Institution must define inclusion and declare itself as inclusive	*“If the university does not state in its navigation chart what is inclusive*, *it is difficult that it may*, *that we might*, *be able to recognize ourselves as an inclusive university and center all necessary efforts to be able to attain it”* [F.G. 2, male executive] *“There must be a clear definition*, *clear lines that set a basic or starting position of the institution”* [I1, female executive]
	The Institution must improve diagnostics and articulate support	*“An inclusive university must know that from the time a student with those characteristics enrolls or applies until he graduates*, *he must have a protocol of how to get in touch*, *to see what are their needs and how we can help”* [F.G. 1, female executive ]
	The Institution must strengthen participation spaces	*“The university itself should make it easy for the person to meet*, *know other realities so that all of us would have an easier life*, *with facilitators*, *not barriers”* [F.G. 2, non-binary student]
	The Institution review sanctions and create protocols for inclusion	*“There are no protocols*, *there is no active mechanism there is no step-by-step*, *nor a regular pathway where the student can notify*, *beyond the teacher or the career head”* [F.G. 1, male student]
	The whole educational community must be educated about inclusion topics	*“It is fundamental that all people belonging to the university community be allowed to learn about this issue [inclusion]*. *To be sincere*, *there is a severe lack of knowledge about this concept and all its implications for the population”* [F.G. 4, male teacher].

### Axial codification

**Fig 1:**
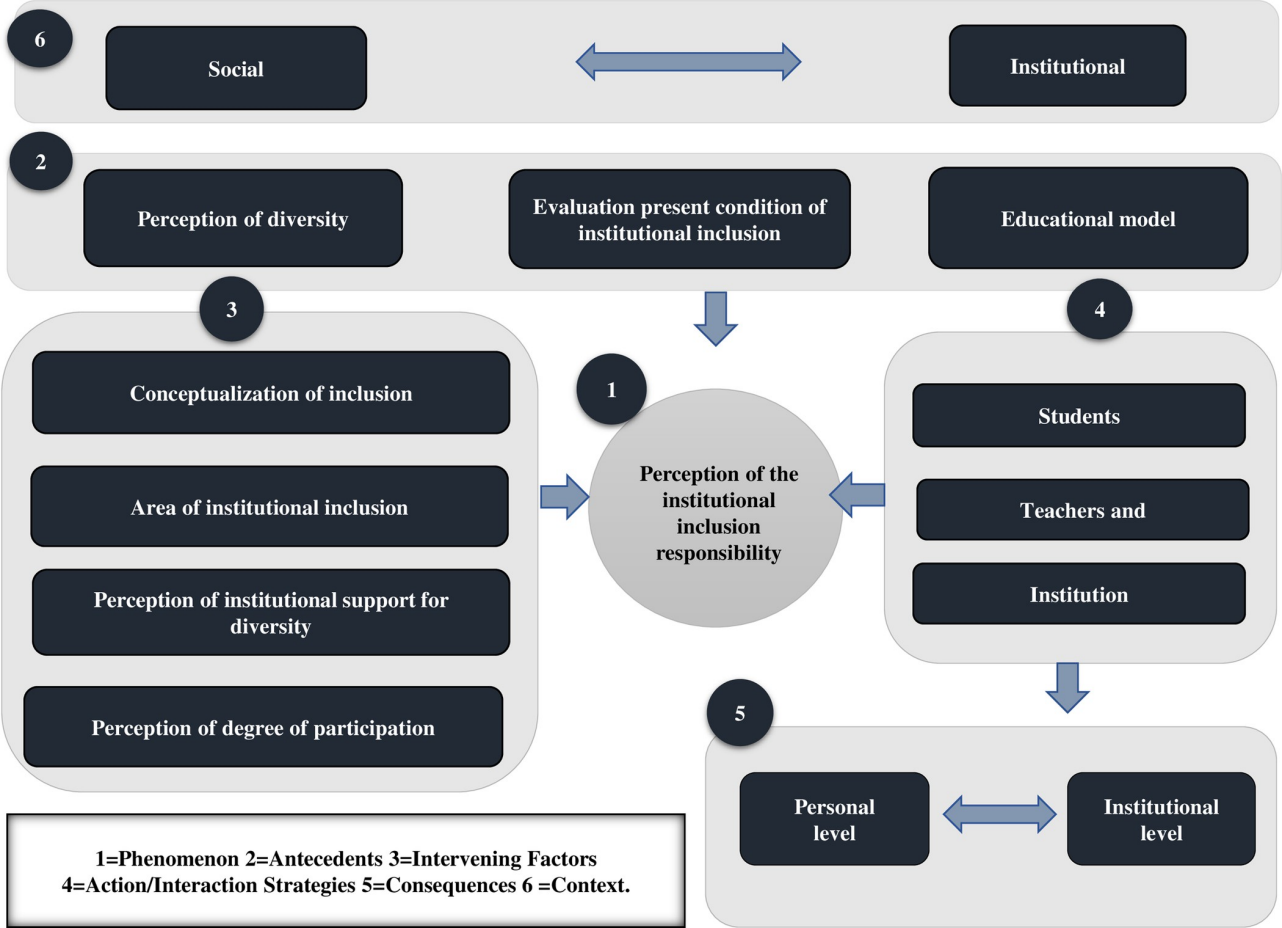
Axial model for perception of the institutional inclusion responsibility.

We built a model (Fig 1), starting from the association between the open codification emerging categories. We based it on the Strauss and Corbin [36] codification paradigm and links context, causal conditions, intervening conditions, action/interaction strategies, and consequences.

#### Context

The perception of the institutional inclusion responsibility phenomenon occurs in a “social context” with several elements that characterize it. Firstly, it is a historical moment in which social movements have allowed the rights of people belonging to groups that have historically been at a disadvantage due to specific characteristics such as sexual orientation, disability, socio-economic vulnerability, belonging to specific ethnic groups, among others, to be visualized and progressively addressed.In the second place, we observed an advance in the norms concerning inclusion and developing public policies focused on rights and social equality in Chile, affirming the value of diversity and recognizing the right to education without discrimination.

The third is the perception that Chilean society is not inclusive and affects interactions at the institutional level. Participants recognize generational differences regarding the acceptance and valuation of diversity, which they perceived as more by younger persons. Furthermore, we observed the opinion that this institution would have a more reactive position when facing socio-cultural requirements and changes, thus requiring taking responsibility and an institutional posture.

On the other hand, the phenomenon takes place in an “institutional context” where the studied University has characteristics that would hinder the possibility of updating its mode of functioning and thought to present social conditions. Among those is a traditional mentality and an elitist origin, remaining from its more than a hundred years of existence, and a structural organization perceived as hierarchic and challenging to modify due to its large size. However, participants mentioned that this situation is changing because the institution is revising constituting elements.

#### Antecedents

Participants observe, especially with students, a highly diverse institution reflected in the existence of gender and sexual, cultural, ethnic, political, religious, and socio-economic diversity and persons in disability situations across the whole university community. At the same time, they observe a trend in the homogenization of personal characteristics. On the other hand, the participants observed the institutional inclusion condition in a development process. Despite not being an inclusive institution, the different support programs and initiatives that promote inclusion show a will to become an inclusive institution.Also, the diagnostic mechanisms for the timely detection of the community members’ needs in the different environments of university life are deficient. Besides, there is no institutional definition of inclusion to provide a consensus for the educational community on the understanding and perspective from which inclusion and the need to rely on an inclusion policy to coordinate, orient, and regulate actions will be addressed. As a final antecedent, students and teachers evaluated the "Educative Model of the University," promoting a teaching-learning process centered on the individual above the collective. They commented that educational processes are difficult to modify when it is required to carry out methodologic adaptations. Also, they observed a teacher role overloaded with tasks, lacking function definitions, and does not state competencies associated with inclusion in its protocols.

#### Intervening factors

Participants have different perceptions of responsibilities on inclusion at a personal, institutional, or social level. They observed differences in responsibilities perceptions according to the understanding of the concept of inclusion. In this way, concerning the inclusion concept, they mentioned feeling ignorant, with scant perspective, doubts, or lacking clarity about the topic. Besides, they mentioned that this concept has yet to be fully integrated and is new for some persons, highly susceptible to changes in time, and quite broad and ambiguous. Likewise, they commented that inclusion has a paternalistic perspective and a reductionist vision of the concept.

They raised the need to broaden the inclusion focus beyond people with disabilities. However, there still needs to be a consensus regarding the university’s ability to respond to the many specific needs of all its members’ communities.

*"The groups with access difficulties are infinite; that is the truth*. *So*, *is the university realistic*? *How far can it take care of everyone*? *[F*.*G*. *3*, *female teache]*.

All the above would make it challenging to assume personal responsibilities, given the feeling of insecurity and the need for clarity regarding the definition and frames of inclusion practices.

*“Then*, *of course*, *it is left to the goodwill of the teacher (…)*, *and I also ask myself*: *up to what point do I go*? *If I am a good pal*, *do I take a further step*? *(…) maybe I am committing an error going further”* [F.G. 1, male teacher].

A second intervening factor is the participant’s concern about admission and infrastructure. They commented that the institution’s responsibility is strengthening contracting to use special enrolling systems for minority groups and structurally adapting the institution to generate inclusion in its community. Also, regarding the teaching-learning and participation process, participants commented on the importance of sharing responsibility between protocols and spaces that the institution generates in favor of inclusion and personal practices of inclusion by all community members.

A third intervening factor is the perception of institutional support for diversity. It influences the perception of the value with which the institution addresses diversities and, therefore, the degree of engagement in response to them and assumes responsibility towards the different groups that make up their educational community. Besides, regular teachers comment about the perception of lacking institutional support regarding the necessary formation of competencies to take care of diversity and the lack of diffused clear protocols to be able to respond to multiple situations requiring special attention. In this way, the overload in the teacher’s role and the excessive number of students per class and functions to be carried out negatively impact the possibility of taking care of the student’s differences.

*“We are told about the advantages of different methodologies to take learning styles into account; however*, *this is very difficult in the University taking into account the number of students per class…”* [G.F. 1, female teacher].

Participants commented on their perception of institutional participation with a general opinion about the University having a more consulting than participating culture. The educational community members refer to being consulted and listened to, not necessarily implying that their opinions affect decision-making. This perception varies according to the position of the person and the group to which the person belongs. In general, this situation would diminish the capability to exercise personal action due to the perception that one’s actions and opinions constitute isolated and disjointed initiatives with no impact at the level of an inclusive organizational culture.

#### Action/interaction strategie

The students take the initiative of assuming the inclusion responsibility using inclusive practices based on accepting and respecting diversities and fostering sensitization and education spaces for diversity and inclusion topics. Moreover, excluding practices related to prejudices and individualism are observed, and participants commented that part of the success of the interactions is related to the degree of knowledge and closeness among peers and diversities.

*“It is understood that they do not know me*, *and I do not know them either*, *but in those cases*, *it is a bit difficult to make people understand that there are things I cannot do and they turn a bit individualistic”* [G.F. 1, student, male].

Regarding the teachers’ and officials’ strategies to face the lack of inclusion protocols, they assume the responsibility of generating support for those who need it. They use personal initiatives and coordinate with career colleagues and faculty regarding methodologic adaptations in the classroom, emotional support, and diagnostic strategies. On the other hand, excluding practices from teachers and officials is associated with deep-seated prejudices and a lack of knowledge on addressing diversity.

*“Then you begin asking for help all over the place*. *Everyone does the best possible and you get a lot of assistance from internal instructions*, *doing all thatcan be done”* [F.G. 1, female teacher].

Finally institutional strategies try to strengthen roles and critical instances to improve diagnostic, intervention, and follow-up of people who need support are:1. The tutor’s system bridges the teacher’s teams and the students to identify and support different academic situations. 2. The level monitors system, which detects difficulties in the student’s teaching-learning processes at the same level and can generate follow-up or derivation to programs/units of the institution. 3. Program for improvement the Career Head has a role as a critical agent for the development and implementation of modifications and improvements. 4. The creation of an early warning system to improve the diagnostics and proceed expeditiously and precisely using the existing support channels and rely on a follow-up along the student’s life.

#### Consequences

Concerning the consequences, we observed at a personal level doubt about one’s capacities to address situations related to inclusion and fear that the actions carried out may be damaging or counterproductive. Teachers highlighted the need for the existence of inclusion protocols and to receive training in this area. Even when they reported that universities are increasingly promoting inclusion, there is still a lack of specific strategies to face with the challenge of build a more inclusive environment.

*“I know*, *especially teachers who have students in disability situations according to the different existing typologies and they point out that they are not able*, *they do not have the specific formation to take care of those students”* [F.G. 1, female executive].

The last situation shows how dealing with situations that require inclusion depends on the will and good intentions of the people involved. Participants feel hindered and insecure by the ability to take action. They need clarity about who to include and how to do so.

*"So*, *of course*, *it is up to the teacher’s will (*…*) and I also ask myself the question*: *How far do I go*? *(*…*) maybe I am making a mistake in going further"* [F.G. 4, male teacher].

From an executive point of view, there is a perception that the student support system is in the process of improvement, as it currently operates in a rudimentary manner and is based on students seeking help directly when they need it.

*"What we do is just to indicate to the faculties and offer students permanently*, *that they also approach when they require certain support*. *So what we do is to welcome students*, *whether they arrive individually or their bosses refer them*, *I don’t know*, *so they are given this type of support or they are referred"* [Interview 1, female executive].

A second observed consequence is the existence of inclusive and excluding practices in the three participant groups. Both situations link with the degree of knowledge and information persons may have about diversity and the degree of closeness with persons with some diversity. Also, this situation is associated with the need for more spaces to favor inclusion.

*“Within the classes schedules*, *there should be another instance where they could get together*, *have some time to know each other*, *but that does not happen*, *and then I feel excluded almost all the time”* [G.F. 1, student, male].

Third, there is a strong perception of images by careers or faculty that students want to conform to, probably due to a feeling of belonging to a group. This situation stifles individual expressions and tends to homogenize by careers or faculties. Participants mentioned that there are more traditional careers, such as health, law, and engineering, where there may be less diversity than in the case of humanistic, social, or educational careers.

In the fourth place, we observed situations of diverse people’s distrust and feared asking for or receiving help. There is the perception that this occurs from fear of rejection and shame. In addition, we observed situations where people who need support do not accept it because they feel positively discriminated against. Participants suggest this has to do with not wanting to recognize themselves as part of a minority in a society that is not inclusive. People commented that they did not perceive this situation as a responsibility of the institution but instead as a result of cultural prejudices associated with diversity and inclusion.

*"(*…*) he did not want any additional help because he felt positively discriminated against*, *because he had hearing problems (*…*) he also had problems with economic resources*. *So*, *we wanted to help him and he did not want help" [F*.*G*. *4*, *female teacher]*.

As a last consequence, at the personal level, participants strongly perceive that it is the responsibility of each community member to perform inclusive practices. This responsibility can show through inclusive language, respectful and collaborative treatment, willingness to help those who need it, recognition of minority groups, and acceptance of diversity.

*“The easiest way of including us is using language*, *which very often is used to mock*, *or like it is something of no account but that it is really (…) necessary*. *To recognize them*, *it is necessary to name things”* [F.G 1, non- binary student].

In addition, there is a perception at the institutional level that, in general, there is a greater openness to diversity than ten or five years ago. Pointing out that the creation of support programs for the existing diversity, the incorporation of diversity content in some curricula, greater visualization expression, and freedom of the diversities are milestones that provide evidence of this change.

*“In the last two or three years*, *sexual diversity content has been incorporated into the syllabus of some subjects”* [F.G. 2, male teacher].

Finally, participants stated a series of challenges from the institution:

- Define inclusion
- Declare itself inclusive
- Improve diagnostics and articulate the already existing support instances
- Strength the participation spaces
- Revise the existing sanctions for discrimination situations
- Create inclusion protocols and educate the community on inclusion topics.

## Discussion

The observed results the role of the social, institutional, and personal environments around the educational inclusion responsibility phenomenon. In this way, the educational community members need to articulate the different levels of institutional action to continue advancing in the project of an institution that recognizes and takes charge of its existing wide diversity. The above follows Booth and Ainscow’s inclusion index [7], which conceive inclusion as a process that implies transforming an educative center’s culture, policies, and practices. Concerning the interaction between the personal and institutional levels observed in this study, we can observe the diffusion of responsibility. Both parts expect that the other should first assume or be considered an inclusive Institution in a determined manner. On the one hand, the community members perceive that it is necessary to strengthen the scope of the policies, declarations, and protocols. It generates an actual sensation of disjointed initiatives or practices that do not achieve a profound impact at the level of organizational culture, whose responsibility is attributed to the Institution. The above would coincide with the findings regarding the inclusion barriers, where the most critical obstacles identified by the students were the negative attitudes shown by Faculty members [26]. On the other hand, we observed that the institution’s strategies strengthened the roles of the educational community members, focusing responsibility at the personal practices level. The above would align with findings in higher education institutions, which indicate that a positive attitude of both students and professors is essential to create favorable inclusive environments and generate a change of mentality towards persons having educational needs [19]. This diffusion of responsibility takes place in a context favored by the ambiguity of the institutional conceptualization of inclusion, which seems to contribute to the perception of non-inclusive climates within the University. Agrees with the statements indicating that there has not been a consensus in the education setting to specify what inclusive education means and what it means to act in an “inclusive” way [41]. It is essential to achieve a consensus on the definition of inclusion, and interviewees state the need to establish the limits to which the institution can act responsibly. To create inclusive educative centers, the members of the communities must have a realistic conceptualization [42]. Given the above, it becomes necessary that the University generates inclusive policies to deliver and orient the essential definitions and strategies within an institutional framework to respond to the diverse needs of the educational community. At the same time, it is essential to stress the need to promote the formation of teachers in competencies associated with inclusion, favor the spaces so that the students may exercise their right to participate in an active and significant way, and finally educate and sensitize the whole educational community about this issue.

At the personal practices level, we identified that each group is responsible for promoting inclusion values within their action limits. It coincides with a revision of the literature concerning the importance of the whole educational community being part of the inclusion process [21]. The inclusive educational praxis is necessary for carrying out methodologic adaptations and internalizing a positive attitude towards inclusion. The students have a vital role in promoting inclusive instances employing respect and acceptance of others in their diversity, and appropriating the participation spaces. Finally, a critical attitude, dialogue, and constant multidirectional feedback among all community members will set the bases for joint work to implement the legal regulations practically.

Based on our results and using the force field model [43], we identify elements that contribute to and hinder the understanding of those who should assume the responsibility of including in the university. The following table (Table 2) details the common aspects that we find in the three groups of people interviewed.

**Table 2:** Driving and restraining forces transversal to a group of students, teachers and non-teaching staff.

Driving Forces	Restraining Forces
Need for an inclusion policy	
Need to generate an institutional definition of inclusion	Lack of an inclusive university policy
Interest in training instances in educational inclusion	Unclear about the conceptualization of inclusion
Interest in generating an action plan that allows realizing inclusive practices in the institution	Lack of spaces in university life to discuss inclusion

There are also driving and restraining forces differentiated according to the group participating in the study. They are detailed below (Table 3).

**Table 3:** Driving and restraining forces by group.

Group	Driving Forces	Restraining Forces
Students	Greater visibility and expression of diversity Positive attitude in acceptance of diversity	Tendency to homogenize personal characteristics by faculty or area of knowledge The tendency of diversities to hide their status for fear of being discriminated against
Teachers	Improvements in diagnosis, intervention and follow-up of students in need of support Growing development of institutional actions to support teachers in favour of inclusion	The feeling of unclarity of the teaching role The feeling of little curricular and methodological flexibility to achieve inclusive classroom practices The difficulty for some faculty members in accepting and understanding student diversity
Non-teaching civil servant	Existence of laws that protect workers with disabilities	Fear of exclusion in the workplace for manifesting diversity

We suggest that future research may delve into the steps that need to be taken by level to achieve an inclusive university.

Regarding the limitations of the study, in the first place, it was carried out in a single Higher Education Institution. Therefore it is necessary to widen it to other learning institutions. Likewise, as to the sample composition, we consider it essential to include more persons belonging to diversities of the three staff and, in the case of the group of teaching officials, be able to reach other positions different from the administration staff.

Finally, this research is a starting point to study the perceptions concerning the phenomenon of responsibility in educational inclusion. Therefore,we suggest that future researches should widen the scope of the different staff and specific dimensions of educational inclusion.

## Supporting information

S1 Table Proposed English translation of Interview topic guide (Directives). (DOCX)

S2 Table Proposed English translation of Interview topic guide (Officials). (DOCX)

S3 Table Proposed English translation of Interview topic guide (Students). (DOCX)

S4 Table Proposed English translation of Interview topic guide (Teachers). (DOCX)

S5 Table Proposed English translation of example of initial coding. (DOCX)
