# Supplementary material for: Inclusion in the university: Who assumes responsibility? A qualitative study
Source: PLoS One. 2023 Jan 20;18(1):e0280161. doi: 10.1371/journal.pone.0280161 (PMC9858406; doi:10.1371/journal.pone.0280161)
Supplement: S1 Table — (DOCX) [file pone.0280161.s001.docx]

| **TOPIC** | **QUESTION** |
| --- | --- |
| Opinions about inclusion | What do you think an inclusive university would look like?  Who should be included and how?  What do you think about the concept of inclusion? do you think it represents those who are excluded?  On whom does it depend for the U to be inclusive and in what way? |
| Inclusion/exclusion experiences | What challenges have you faced in becoming a executive or holding the position you have?  Mention a time when you have and have not felt included.  Comment an experience in which a particular person or group has been labeled/stereotyped. How has that situation been handled? |
| Institutional support | Mention an experience in which you have needed some type of support. To whom did you turn to? how was that experience?  If you have experienced any situation of discrimination or intimidation, how did the institution react?  Do you feel your opinions are heard and generate the expected changes? |
| Diversity perception | What kind of diversity is there in the executive team?  Do you think the positions on the executive team reflect the balance of gender and other groups present at the U?  What kind of diversity is there at the university in general? |
| Diversity valuation | If you had to propose instances or practices that would allow reinforcing the value of each person within the institution, what would they be? |
| Ending question | In relation to all that was discussed, would you consider this university is inclusive? |

S1. Proposed English translation of Interview topic guide (Directives)
