# Supplementary material for: Inclusion in the university: Who assumes responsibility? A qualitative study
Source: PLoS One. 2023 Jan 20;18(1):e0280161. doi: 10.1371/journal.pone.0280161 (PMC9858406; doi:10.1371/journal.pone.0280161)
Supplement: S2 Table — (DOCX) [file pone.0280161.s002.docx]

| **TOPIC** | **QUESTION** |
| --- | --- |
| Opinions about inclusion | What do you think an inclusive university would look like?  Who should be included and how?  What do you think about the concept of inclusion? do you think it represents those who are excluded?  On whom does it depend for the U to be inclusive and in what way? |
| Inclusion/exclusion experiences | Mention a time when you have and have not felt included.  Comment an experience in which a particular person or group has been  labeled/stereotyped. How has that situation been handled? |
| Relationships | What type of work experience is more common in your day-to-day work: supportive/cooperative or competitive/individualistic? |
| Institutional support | Mention an experience in which you have needed some type of support. To whom did you turn to? how was that experience?  If you have experienced any situation of discrimination or intimidation, how did the institution react?  Do you feel your opinions are heard and generate the expected changes? |
| Diversity perception | Are officials diverse? How do you observe this?  Do you consider this U to be a diverse university and in what way?  Comment on any negative or positive experience relating to a historically segregated group or group different from your own. |
| Diversity valuation | If you had to propose instances or practices that would allow reinforcing the value of each person within the institution, what would they be? |
| Ending question | In relation to all that was discussed, would you consider this university is inclusive? |

S2. Proposed English translation of Interview topic guide (Executives)
