# Supplementary material for: Inclusion in the university: Who assumes responsibility? A qualitative study
Source: PLoS One. 2023 Jan 20;18(1):e0280161. doi: 10.1371/journal.pone.0280161 (PMC9858406; doi:10.1371/journal.pone.0280161)
Supplement: S3 Table — (DOCX) [file pone.0280161.s003.docx]

S3. Proposed English translation of Interview topic guide (Students)

| **TOPIC** | **QUESTION** |
| --- | --- |
| Opinions about inclusion | What do you think an inclusive university would be like?  Who should be included and in what way?  What do you think about the concept of inclusion, do you think it represents those who are excluded?  On whom does it depend that the U is inclusive and in what way? |
| Inclusion/exclusion experiences | Mention a time when you have not felt (and times when you have felt) included.  Comment an experience in which a particular person or group has been labeled/stereotyped. How has that situation been handled? |
| Teaching and learning process | Do they think their teachers expect the same from all their peers or who do they expect more from?  Comment on a situation in which you felt you were able to reach your greatest potential as a student, what role did the institution play there? |
| Institutional support | Mention an experience in which you have needed some type of support. To whom did you turn to? how was that experience?  If you have experienced any situation of discrimination or intimidation, how did the institution react?  Do you feel your opinions are heard and generate the expected changes? |
| Diversity perception | Are student diverse? In what way?  Do you consider this U to be a diverse university and in what way?  Comment on any negative or positive experience relating to a historically segregated group or group different from your own |
| Diversity valuation | If you had to propose instances or practices to reinforce the value of each person within the institution, what would they be? |
| Ending question | In relation to all that was discussed, would you consider this university is inclusive? |
