# Supplementary material for: Inclusion in the university: Who assumes responsibility? A qualitative study
Source: PLoS One. 2023 Jan 20;18(1):e0280161. doi: 10.1371/journal.pone.0280161 (PMC9858406; doi:10.1371/journal.pone.0280161)
Supplement: S5 Table — (DOCX) [file pone.0280161.s005.docx]

S5. Proposed English translation of example of initial coding

| **Interview statement** | **Topic** | **Code** | **Category** |
| --- | --- | --- | --- |
| ...The issue of educating in respect, in tolerance, in some values that allow us to be more inclusive at all levels. But I think we have to educate the entire university community, both male and female officials. Where we are all on the same page and it is not "the cases of disability we refer them to the group that sees disability" it has to be a known policy and where we all manage what is done and how to approach this issue, which as I say not only/ I see that it would be necessary to join efforts because, for example [University´s program for inclusion] today works with disability, but the visual disability is worked by [University’s program for visual disability], but the gender issue is worked by the [University’s department for gender issue] but the ethnic issue is not focused in any department…so it is kind of disintegrated and I think that, in order to make an institutional policy, it would have to join efforts, first define what inclusion is, what we are going to understand, how we are going to work on it and something much more macro, I think. And education above all, for everyone. | I believe it is necessary to educate in values that promote inclusion.  I believe that it is necessary to educate the entire university community and unify the institution's vision around inclusion.  I think that the institution's vision of inclusion should be made known and this information should be extended to the entire educational community.  I think that at present the work with inclusion is disintegrated by units and it is necessary to unify efforts in an institutional policy of inclusion.  I believe that the institutional policy must define what inclusion is and how it will work, from a macro perspective, including education for the whole community. | It is necessary that the  whole educational  community be educated  about inclusion topics  It is necessary that the  Institution should define  inclusion and declare itself  as inclusive | Challenges to  generate an inclusive  Institution |
|  |  |  |  |
